# Effect of fracture level on the residual fracture gap during tibial intramedullary nailing for tibial shaft fractures

**DOI:** 10.1051/sicotj/2023023

**Published:** 2023-08-11

**Authors:** Kutalmis Albayrak, Abdulhamit Misir, Yakup Alpay, Abdul Fettah Buyuk, Evren Akpinar, Sukru Sarper Gursu

**Affiliations:** 1 University of Health Sciences Baltalimani Bone Diseases Training and Research Hospital, Department of Orthopaedics and Traumatology 34470 Istanbul Turkey; 2 Private Safa Hospital, Department of Orthopaedics and Traumatology 34194 Istanbul Turkey; 3 Bahçeşehir University, Vm Medical Park Maltepe Hospital 34846 Istanbul Turkey; 4 University of Missouri, Department of Orthopaedics Columbia MO 65201 USA; 5 Professor, University of Health Sciences Baltalimani Bone Diseases Training and Research Hospital, Department of Orthopaedics and Traumatology 34470 Istanbul Turkey

**Keywords:** Tibia shaft fractures, Fracture gap, Intramedullary nailing, Union, Fracture level

## Abstract

*Introduction*: The development of fracture gap during intramedullary nailing in tibial fractures is associated with poor fracture fragment contact and increased time to union and complications. This study aimed to evaluate the effect of the fracture level in the development of the fracture gap and the effect of the fracture gap on pain, radiologic and functional outcomes, and complication rate. *Material and method*: A total of 45 patients who underwent reamed intramedullary nailing due to closed transverse or short oblique tibial shaft fractures were divided into the proximal fracture group and the distal fracture group. The correlations between the visual analog scale (VAS) score, modified radiograph union score for tibias (RUST), and postoperative 1-year lower extremity functional scale scores, residual fracture gap, and time to union were evaluated. *Results*: The mean fracture gap amounts in the immediate postoperative anteroposterior and lateral radiographs were 5.6 ± 1.7 and 6.0 ± 1.7 mm in proximal fractures and 0.3 ± 2.4 mm and 0.4 ± 2.3 mm in distal fractures, respectively (*p* < 0.001 and *p* < 0.001, respectively). The mean time to union was 21.9 ± 2.9 weeks in the proximal fracture group and 16.7 ± 2.4 weeks in the distal fracture group (*p* < 0.000). The residual fracture gap amount significantly correlated with the level of fracture (*r* = 0.811, *p* < 0.001). *Discussion*: Tibial shaft fractures proximal to the isthmus level tend to develop significantly larger fracture gaps than distal fractures. It is associated with increased time to union and radiographic union scores as well as slightly higher complication and reoperation rates.

## Introduction

Proximal third, mid-shaft and distal third tibial shaft fractures can be treated with intramedullary nailing [1]. Currently, after closed reduction, inserting the intramedullary nail with reaming is more commonly used than with the un-reamed technique [2].

In contrast to mid-shaft fractures, proximal and distal third fractures are prone to difficulties in reduction and malalignment during and after intramedullary nailing [3]. Several techniques have been developed to solve these problems [3]. With the use of interlocking intramedullary nails, in addition to increased stability, loss of length and rotational problems have been solved [4]. However, in spite of the advantages of employing locking nails to maintain reduction, challenges related to nonunion and delayed union persist in fractures that have not been adequately reduced, particularly those displaying an abnormal “fracture gap” [5]. Notably, the presence of a fracture gap constitutes a significant contributing factor to the unresolved issues of nonunion and delayed union [6].

A fracture gap causes poor contact at the fracture site [7]. The size of the fracture gap increases the risk of nonunion development [8, 9]. A fracture gap may develop because of soft tissue interposition, malalignment of the fracture fragments, bone loss, and distraction of the fracture fragments [10, 11].

**Figure 1:**
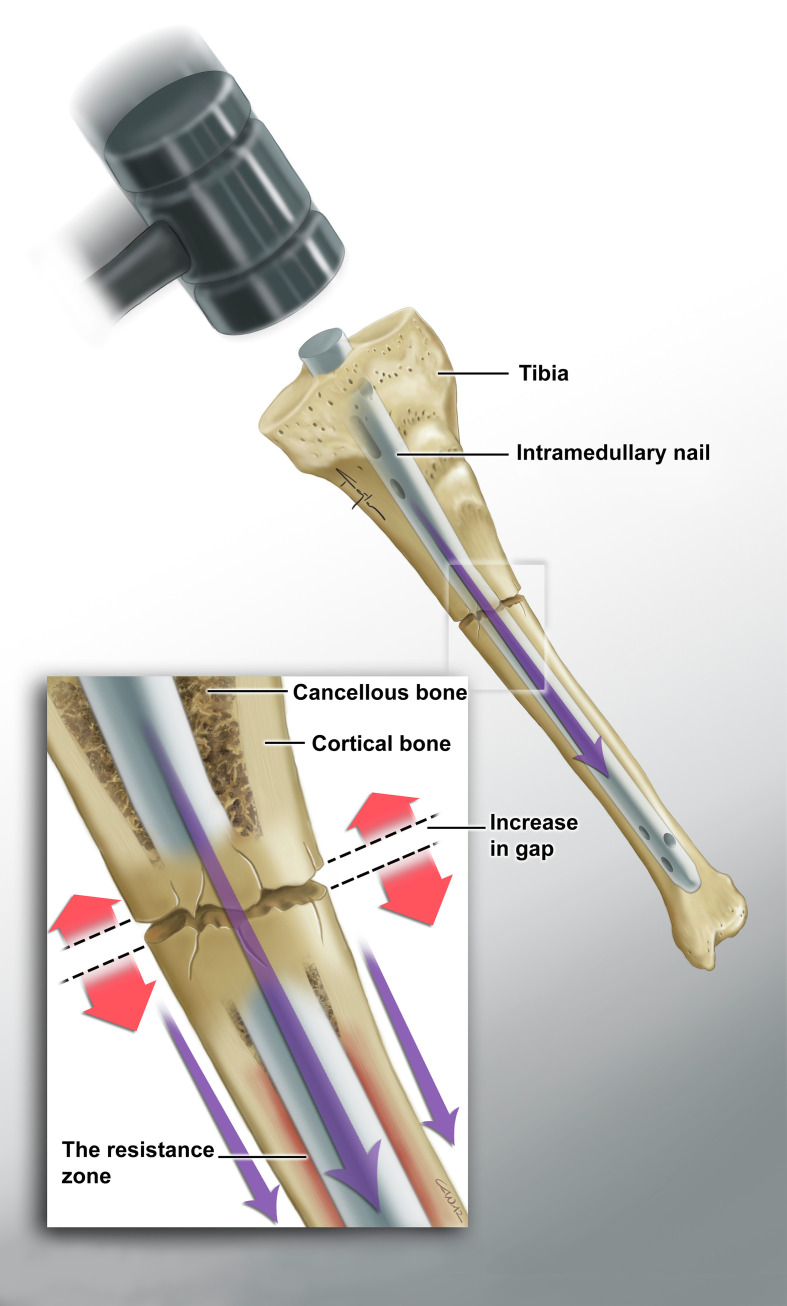
Medical illustrations showing the effect of fracture levels on fracture gap during nailing in tibial fractures, proximal fracture.

**Figure 2:**
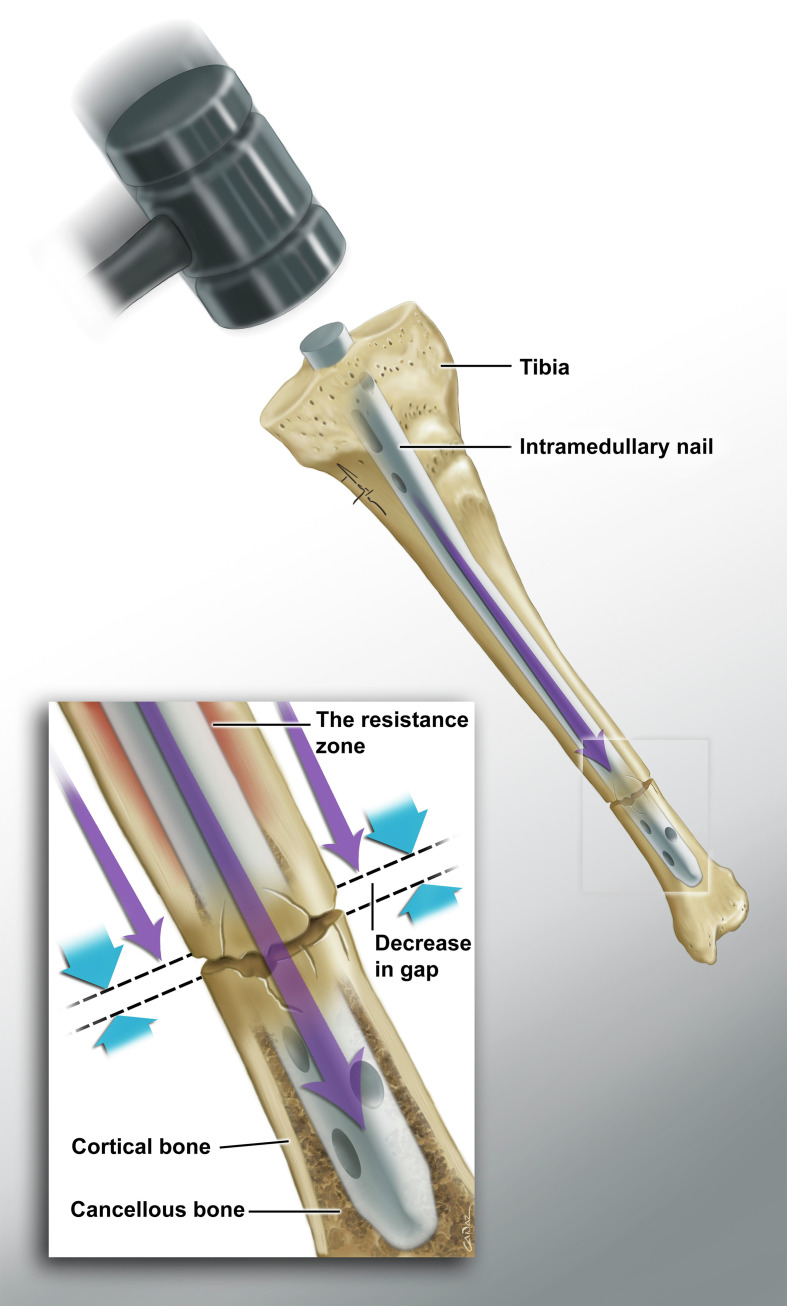
Medical illustrations showing the effect of fracture levels on fracture gap during nailing in tibial fractures, distal fracture.

It is recommended to reduce the fracture gap as much as possible in intramedullary nailing [12]. However, fracture gaps may develop during nailing in some cases. Tibial intramedullary nailing can create a distraction effect according to the fracture level when fitting a nail of appropriate thickness at the isthmus level (the middle 1/3 of the tibial shaft, where the intramedullary nail is in most contact with the bone cortex) as it passes from the proximal to distal fragment. Inversely, in fractures distal to the isthmus, the nail stuck in the bone at the level of the isthmus has a reducing effect on the fracture gap during the hammering (Figures 1 and 2) [13].

To the best of our knowledge, no studies have evaluated the effect of the fracture level on the development of fracture gaps during tibial intramedullary nailing. Thus, this retrospective study aimed to investigate the effect of fracture level (proximal and distal of isthmus level) on the fracture gap during tibial intramedullary nailing in transverse and short oblique fracture types. We hypothesized that the fracture level proximal to the isthmus was associated with an increased residual fracture gap.

## Patients and methods

### Ethical information

The study protocol was approved by the Institutional Review Board (approval date and number: (08.06.2018/30). Informed consent was obtained from all patients.

### Patients

**Figure 3:**
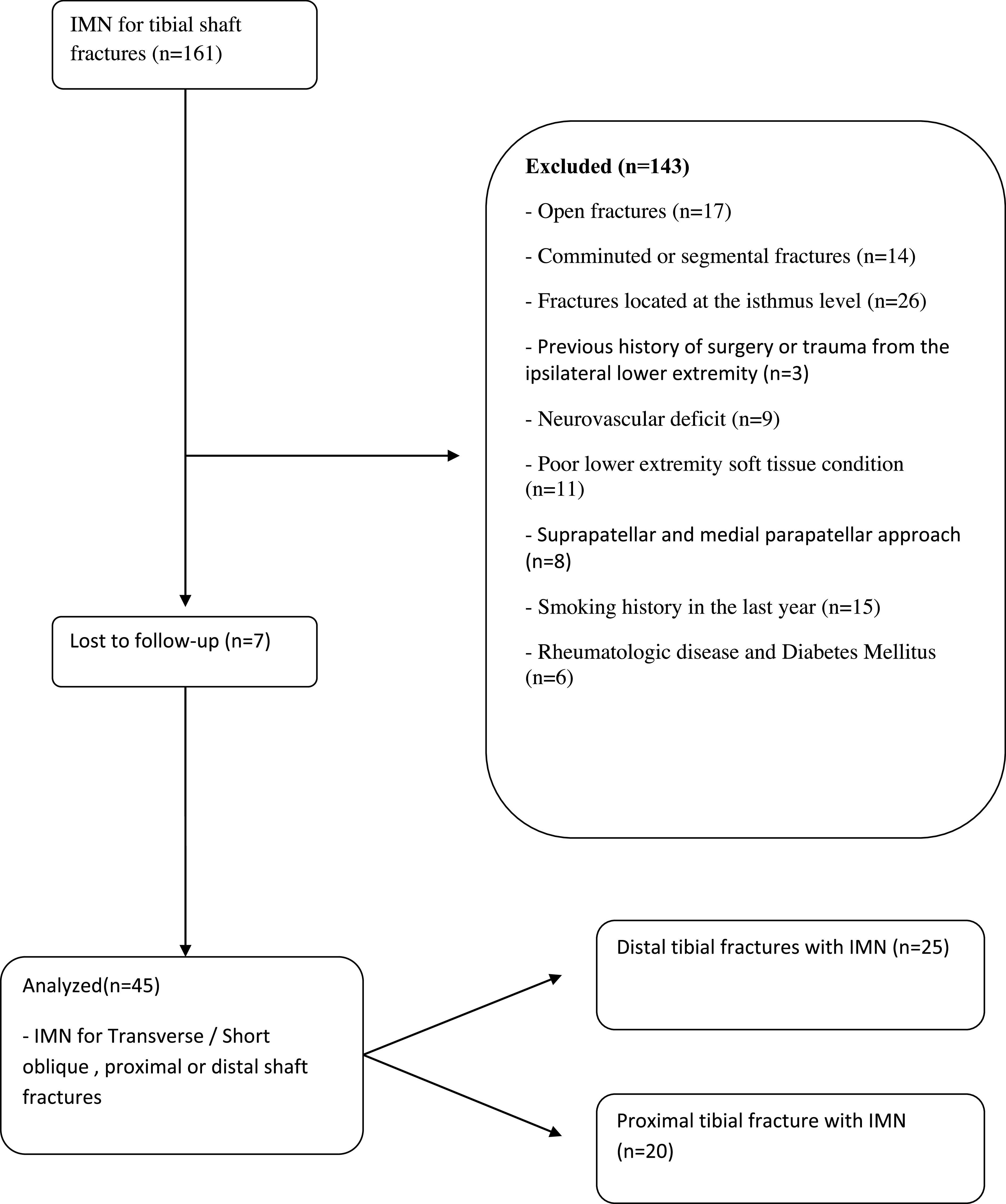
X-ray images of an exemplary case showing residual fracture gap in fractures proximal to the isthmus.

**Figure 4:**
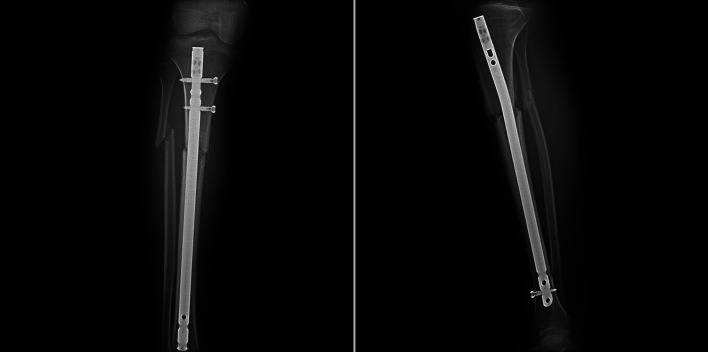
X-ray images of an exemplary case of fractures distal to the isthmus.

In this retrospective study, tibial shaft fractures operated between 2014 and 2016 were analyzed. Between 2014 and 2016, 161 patients underwent tibial intramedullary nailing for unilateral tibial shaft fractures. Patients with tibia shaft fractures proximal and distal to the isthmus level, simple transverse or short oblique fracture pattern, and aged 18–60 years were included. Strict exclusion criteria were applied to homogenize the study group as much as possible. Our exclusion criteria were; (1) open fractures, (2) fractures at the level of the isthmus, (3) comminuted or segmental fractures, (4) neurovascular deficit, (5) history of previous surgery on the same extremity, (6) poor soft tissue coverage, (7) suprapatellar approach, (8) history of smoking, (9) history of rheumatologic disease or diabetes, (10) patients lost to follow-up. After exclusions, 45 patients were included in the study. Patients were divided into two groups based on the fracture level: proximal to the isthmus (proximal fracture group, *n* = 20) and distal to the isthmus (distal fracture group, *n* = 25). Separation of more than 2 mm between fracture lines at the anteroposterior (AP) or lateral radiographs was defined as a fracture gap (Figures 3 and 4). The isthmus region has a narrow and uniform morphology, unlike the remaining tibial anatomy that expands proximally and distally, and distraction or compression is not expected when the nail is advanced from the proximal fragment to the distal fragment [14]. Therefore, fractures within the isthmus region were excluded.

### Data collection

Preoperative patient characteristics including age, sex, bodyside, body mass index, trauma mechanism, concomitant injuries, time from injury to surgery, and comorbidities were recorded and compared between the proximal and distal fracture groups. The type of fracture (transverse or short oblique), fracture localization (proximal third or distal third), nail diameter, nail length, postoperative gap amount, time to union, presence of concomitant fibular fracture, angulation between proximal and distal fragments, development of malunion or nonunion, and distance between the fracture and isthmus were measured and evaluated. Measurements were performed monthly using preoperative and postoperative 1-, 2-, 3-, and 4-month and final (>6 months) follow-up tibial radiographs by two orthopedic surgeons retrospectively who were not involved in the treatment and follow-up of patients and blinded to patient data. The first postoperative X-ray was used for residual fracture gap measurement. Radiologic measurements were performed using the Infinitt PACS System (Infinitt Healthcare Co., Seoul, South Korea) with an accuracy of 0.1 mm for the linear measurements and 0.1° for the angular measurements.

The modified radiographic union scale for tibial fractures (RUST) score was used in the evaluation of radiologic fracture union on postoperative 0-, 1-, 2-, 3-, and 4-month follow-up radiographs [14, 15]. Postoperative 0-, 1-, 2-, 3- and 4-month visual analog scale (VAS) scores and postoperative 1-year follow-up lower extremity functional scale scores were used in the evaluation of pain and functional outcome of patients [16]. The lower extremity functional scale consists of 20 questions, where each question is scored between 0 and 4 points. The percentage of the score obtained over a total of 80 points is calculated. Higher scores indicate better function. Postoperative complications were compared between the two groups.

### Surgical technique

All surgical procedures were performed by an experienced orthopedic trauma team within 7 days after the initial trauma and under spinal anesthesia. Patients were positioned supine. Under fluoroscopic guidance, all fractures were reduced manually. A guide wire was inserted through the medial parapatellar approach and advanced until 2 cm proximal to the ankle joint while the knee was in 90–110° flexion. Then, reaming was performed using appropriate reamers which were determined preoperatively according to the AP and lateral intramedullary canal diameters at the isthmus level. A tibial intramedullary nail with appropriate length and size was inserted. After the distal locking screws were placed, two or three proximal locking screws were placed after the back-hammering was applied to prevent the fracture gap. All of the fractures were tibia shaft fractures, so there was no need to use a blocking screw. Metaphyseal fractures were excluded from the study.

### Statistical analysis

The mean, standard deviation, median, lowest and highest values, frequency, and ratio values were used in the descriptive statistics of data. The distribution of variables was determined using the Kolmogorov–Smirnov test. The independent samples *t*-test and Mann–Whitney *U* test were used to analyze independent quantitative data, and the chi-square test and Fisher exact test were used to analyze independent qualitative data. The Spearman and Pearson correlation analyses were used in the evaluation of the correlation between fracture and patient characteristics, and of the fracture gap amount. Intraclass correlation coefficient (ICC) and Fleiss kappa (*κ*) coefficient were used in the evaluation of intra- and interobserver agreement of measurement parameters and union decision. The agreement for ICC and *κ* were defined as follows: 0–0.20, slight agreement; 0.21–0.40, fair agreement; 0.41–0.60, moderate agreement; 0.61–0.8, substantial agreement; and >0.81 as perfect agreement [16, 17]. A *p*-value of <0.05 was considered statistically significant. SPSS version 22.0 (IBM Corp., Armonk, NY) was used in all statistical analyses.

## Results

**Figure 5:**
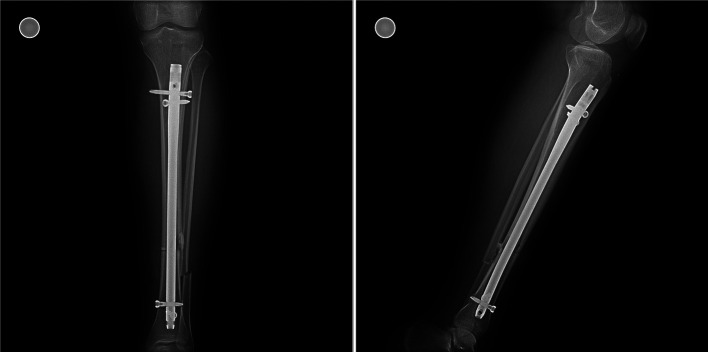
Study flowchart.

The study flowchart is presented in Figure 5. One hundred and sixty-one patients underwent tibial intramedullary nailing for tibial shaft fractures. Patients with open fractures (*n* = 17), fractures located at the isthmus level (*n* = 26), comminuted or segmental fractures (*n* = 14), neurovascular deficit (*n* = 9), previous history of surgery or trauma from the ipsilateral lower extremity (*n* = 3), poor lower extremity soft tissue condition (*n* = 11), suprapatellar approach (*n* = 8), smoking history in the last 1 year (*n* = 15), and rheumatologic disease history or diabetes (*n* = 6) and patients lost to follow-up (*n* = 7) were excluded. No significant difference in patient characteristics was found between proximal and distal fractures (*p* < 0.05) (Table 1). There was perfect intra- (ICC = 0.88) and interobserver (ICC = 0.82) agreement for modified RUST scores obtained using postoperative 1-, 2-, 3- and 4-month follow-up radiographs and fracture union decision (*κ* = 0.90).

**Table 1:** Patient characteristics.

	All patients (*n* = 45)	Proximal fractures (*n* = 20)	Distal fractures (*n* = 25)	*P* values
Age (years)	28.1 ± 12.3	28.6 ± 10.2	27.5 ± 11.6	0.764
Sex				
Female	19 (42.3%)	10 (50%)	9 (36%)	0.165
Male	26 (57.7%)	10 (50%)	16 (64%)	
BMI (kg/m^2^)	23.7 ± 2.5	23.8 ± 2.6	23.6 ± 2.4	0.731
Side				
Right	28 (62.3%)	13 (65%)	15 (60%)	0.239
Left	17 (37.7%)	7 (35%)	10 (40%)	
Fracture type				
Transverse	20 (44.5%)	11 (55%)	9 (36%)	0.331
Oblique	25 (55.5%)	9 (45%)	16 (64%)	
Fibula fracture				
Yes	39 (86.7%)	16 (80%:)	23 (92%)	0.231
No	6 (13.3%)	4 (20%)	2 (8%)	
Nail size (mm)	10.8 ± 1.3	10.9 ± 1.4	10.8 ± 1.2	0.665
Follow-up period (months)	34.0 ± 17.5	33.1 ± 19.3	34.7 ± 16.3	0.764
Time to union (weeks)	19.0 ± 3.7	21.9 ± 2.9	16.7 ± 2.3	**0.000**
Fracture-isthmus length (mm)	45.8 ± 25.5	40.5 ± 12.6	52.6 ± 34.9	0.114
Residual fracture gap (AP) (mm)	2.6 ± 3.4	5.6 ± 1.6	0.3 ± 2.4	**0.000**
Residual fracture gap (Lat) (mm)	2.8 ± 3.5	6.0 ± 1.7	0.4 ± 2.2	**0.000**

Bold values indicate statistical significance.

The mean fracture gap amounts in the immediate postoperative AP and lateral radiographs were 5.6 ± 1.7 and 6.0 ± 1.7 mm (average, 5.8 ± 1.6 mm) in the proximal fractures and 0.3 ± 2.4 mm and 0.4 ± 2.3 mm (average, 0.3 ± 2.3 mm) in distal fractures, respectively (*p* < 0.001 and *p* < 0.001, respectively) (Table 1). The mean time to union was 21.9 ± 2.9 weeks in the proximal fracture group and 16.7 ± 2.4 weeks in the distal fracture group (*p* < 0.001). The amount of fracture gap was significantly correlated with the fracture level (*r* = 0.811, *p* < 0.001). A significant difference was found between the two groups regarding postoperative 2-, 3-, and 4-month modified RUST scores (Table 2). The lower extremity functional scale score at postoperative 1 year was not significantly different between the two groups (74.1 ± 7.6 vs. 76.8 ± 3.0, *p* = 0.149). No significant difference was noted between male and female patients and transverse and oblique fractures regarding any parameters evaluated (*p* > 0.05).

**Table 2:** Postoperative changes in mRUST and VAS scores.

	Proximal fracture (*n* = 20)	Distal fracture (*n* = 25)	*P* values
Modified RUST score			
1 month	4.1 ± 0.3	4.5 ± 1.0	0.038
2 months	5.5 ± 1.4	6.7 ± 2.0	0.032
3 months	7.6 ± 2.4	10.1 ± 1.9	0.000
4 months	9.8 ± 2.9	13.9 ± 2.2	0.000
Final score	14.6 ± 3.6	16 ± 0	0.063
Visual analogue scale			
First day postoperative	3.6 ± 1.6	3.8 ± 1.9	0.667
1 month	2.8 ± 1.9	2.7 ± 1.9	0.825
2 months	2.2 ± 1.9	2.1 ± 1.8	0.819
3 months	2.1 ± 1.7	1.4 ± 1.3	0.137
Final score	1.0 ± 1.5	0.3 ± 0.7	0.106

The delayed union developed in four patients (20%) in the proximal fracture group. The gap amount in fractures with delayed union was 5.36 mm and 5.60 mm. After dynamization at postoperative 16 weeks, two fractures were united. Nonunion developed in two patients (10%) in the proximal fracture group. The amount of gap in fractures with nonunion was 7.33 mm and 7.83 mm. Exchange nailing and fibular osteotomy were performed in both patients, and union was achieved 5–6 months postoperatively. There was no delayed union or nonunion in any patient in the distal fracture group. Three patients (one patient (5%) in the proximal fracture and two patients (8%) in the distal fracture group, *p* = 0.784) complained of screw head prominence in the distal medial screws. In these patients, prominent screws were removed at postoperative 6–9 months.

## Discussion

To the best of our knowledge, this is the first study evaluating the effect of fracture level on the development of fracture gap during intramedullary nailing of the tibia. The most important findings of this study were as follows: Although all closed techniques such as back-hammering(backslap technique) after locking have been tried, in tibial shaft fractures located proximal to the isthmus, significant residual fracture gap develops during intramedullary nailing when compared with distal fractures. That increased fracture gap was associated with a longer time to union, worse radiographic union scale scores, and slightly higher complication and reoperation rates.

This study has some limitations. First, the sample size was relatively low. This is because the trauma volume of the center where the study was conducted was not high during the study period. The study of the subject could be more objective with a prospectively designed study. Second, although, all of the cases were performed by senior trauma surgeons in the same clinic and with the same surgical protocol, it is a limitation that it was not performed by a single surgeon. Third, quality-of-life measurement tool such as Short Form-36 was not used in the evaluation of patients. It would have been useful in revealing differences in patients’ quality of life. Fourth, distal and proximal fractures will have different union biology and duration due to their inherent nature. After intramedullary nailing of tibial fractures, a study has shown that functional and quality of life may differ within the first 1 year and after 1 year [18].

Fracture healing is affected by the mechanical environment. Gap size and interfragmentary strain were identified as mechanical factors for fracture healing [19]. Various studies have shown that a larger interfragmentary gap is associated with less callus formation, less vascularization, more fibrocartilage tissue formation, reduced interfragmentary strain, and delayed healing [11, 20, 21]. In clinical practice, although surgeons are aware that large fracture gaps tend to be less likely to heal, the exact gap size that requires further intervention is not well known. Gaebler et al. reported that a fracture gap > 3 mm increases the risk of delayed union by 12 times and nonunion by four times [22]. Salem et al. reported that the fracture gap significantly affects the union time [23]. They reported a mean union time of 29.5 weeks in fractures with fracture gap > 5 mm, while it was 17 weeks in fractures with compression. In addition, fractures having comminution in addition to the fracture gap had a mean union time of 34 weeks. They also showed 1.6 times increased risk of delayed union and nonunion in cases with 1–3 mm of the gap at the fracture site, while six times increased risk in cases with >3 mm of fracture gap. Drosos et al. reported an increased risk of nonunion in cases with fracture gap ≥ 3 mm in tibial diaphyseal fractures without or minimal comminution [12]. In the present study, the fractures located proximal to the isthmus level tend to develop a fracture gap during intramedullary nailing. The residual fracture gap was associated with longer time to union, radiological union scores, and increased delayed union and nonunion rates.

Many techniques have been described in the literature to prevent residual fracture gaps in tibial fractures. However, if this issue is not paid attention in clinical practice, it will remain a problem in proximal tibial shaft fractures. In our opinion, the back-hammering technique after distal locking is not sufficient to prevent a fracture gap. Using larger reamers to prevent the nail from getting stuck in the isthmus of the tibia during nail placement is effective in solving the problem, especially in proximal tibial fractures.

As we tried to demonstrate in Figures 1 and 2, if a sufficiently wide reaming procedure is not performed, an unavoidable fracture gap will occur due to the resistance formed between the nail and the bone cortex during nail placement in proximal tibial shaft fractures. However, since this resistance zone remains proximal to the fracture line in distal fractures, it will have an effect on reducing the fracture gap during nail placement.

Results regarding the relationship between the level of diaphysis fracture and time to union are controversial. Gaston et al. reported that proximal fractures have a slower time to union than middle and distal fractures [24]. However, Drosos et al. showed no association between union time and fracture level [12]. Although the literature indicates that proximal tibial fractures are more prone to nonunion and malunion, it is the metaphyseal fractures of the proximal tibia that are mentioned in the literature. Certainly, there are problems related to both reduction and fixation stability. However, in our study, it is not the metaphyseal fractures but the problems in proximal tibial shaft fractures that we tried to demonstrate. In our study, the mean time to union was significantly longer in fractures located proximal to the isthmus. We attribute that to the significantly increased fracture gap in proximal fractures.

The modified RUST was developed to assess the healing of tibial shaft fractures after intramedullary nailing [25]. It is a reliable tool and widely used in the evaluation of radiographic tibial fracture union [26]. In cases with a wide fracture gap, bony bridging by hard callus cannot be obtained despite the presence of good callus formation, and delayed union or nonunion may develop. In addition, stiff fixation and wide gap create a low-strain environment which predisposes to delayed union or nonunion [21]. All of these conditions affect the modified RUST score and union decision in the specified timeframe. In the present study, a significant difference was observed between the modified RUST scores of proximal and distal fractures in all time points. It was attributed to the lower callus amount and sustained fracture line visibility related to the increased gap in proximal fractures.

Despite being a subjective parameter, pain at the fracture site in either palpation or weight-bearing is a good clinical indicator of fracture healing when combined with radiologic methods [27]. Pain persists with delayed union [28]. However, in our study, we found slightly but not significantly higher VAS scores in patients with a proximal fracture at all postoperative time points. Certainly, this cannot be entirely attributed to the increased residual fracture gap, but its effect should be discussed.

Good functional outcomes with relatively high complication and reoperation rates have been reported after intramedullary nailing of tibial shaft fractures. Reoperation rates after intramedullary nailing of long bone fractures ranged from 12% and 44%. Identifying the factors that cause poor outcomes is important for patient care [29]. Several factors, such as age, sex, injury mechanism, severity of tissue damage, fracture morphology, time of surgery, diabetes, smoking, vasculopathy, alcohol use, and corticosteroids, were associated with negative outcomes and have been evaluated in multiple studies [30]. In their prospective multicenter study, Schemitsch et al. reported that the fracture level was not a factor that increased the risk of negative events. However, a fracture gap < 1 cm has been associated with a 2.4-fold increased risk of negative events when compared with no fracture gap. Similarly, Bhandari et al. showed that the fracture gap, transverse fracture pattern, and open fracture were determined as predictors of reoperation [31]. In our study, delayed union and nonunion developed in four patients (20%, two delayed union, and two nonunion) with proximal fractures. However, none of the patients in the distal fracture group developed delayed or nonunion.

## Conclusion

Although many reduction methods are used to reduce fracture during the nailing of tibial shaft fractures, tibial shaft fractures proximal to the level of the isthmus tend to develop a significantly larger fracture gap than distal fractures. It is associated with increased time to union, radiographic union scores, and slightly higher complication and reoperation rates.

## Data Availability

The data and materials used in this study are available upon request from the corresponding author.
